# Effect of Chemical Composition on the Thermoplastic Formability and Nanoindentation of Ti-Based Bulk Metallic Glasses

**DOI:** 10.3390/ma17071699

**Published:** 2024-04-08

**Authors:** Mengliang Chen, Liu Zhu, Yingwei Chen, Sheng Dai, Qijie Liu, Na Xue, Weiwei Li, Jinfang Wang, Yingqi Huang, Kaice Yang, Ling Shao

**Affiliations:** 1 School of Materials Science and Engineering, Zhejiang Sci-Tech University, Hangzhou 310018, China; 2 Zhejiang Provincial Key Laboratory for Cutting Tools, Taizhou University, Taizhou 318000, China; sdai@tzc.edu.cn (S.D.); xuena@tzc.edu.cn (N.X.); lww@tzc.edu.cn (W.L.); wjf0909@tzc.edu.cn (J.W.); yingqihuang77@hotmail.com (Y.H.); 17338523070@163.com (K.Y.); 3 Taizhou Key Laboratory of Medical Devices and Advanced Materials, Research Institute of Zhejiang University-Taizhou, Taizhou 318000, China; cywsn@163.com (Y.C.); tanksman@163.com (Q.L.)

**Keywords:** Ti-based bulk metallic glass, viscosity, thermoplastic formability, nanoindentation, average atomic distance

## Abstract

A series of Ti_41_Zr_25_Be_34-*x*_Ni*_x_* (*x* = 4, 6, 8, 10 at.%) and Ti_41_Zr_25_Be_34-*x*_Cu*_x_* (*x* = 4, 6, 8 at.%) bulk metallic glasses were investigated to examine the influence of Ni and Cu content on the viscosity, thermoplastic formability, and nanoindentation of Ti-based bulk metallic glasses. The results demonstrate that Ti_41_Zr_25_Be_30_Ni_4_ and Ti_41_Zr_25_Be_26_Cu_8_ amorphous alloys have superior thermoplastic formability among the Ti_41_Zr_25_Be_34-*x*_Ni*_x_* and Ti_41_Zr_25_Be_34-*x*_Cu*_x_* amorphous alloys due to their low viscosity in the supercooled liquid region and wider supercooled liquid region. The hardness and modulus exhibit obvious variations with increasing Ni and Cu content in Ti-based bulk metallic glasses, which can be attributed to alterations in atomic density. Optimal amounts of Ni and Cu in Ti-based bulk metallic glasses enhance thermoplastic formability and mechanical properties. The influence of Ni and Cu content on the hardness of Ti-based bulk metallic glasses is discussed from the perspective of the mean atomic distance.

## 1. Introduction

Bulk metallic glasses (BMGs) exhibit remarkable mechanical properties and superb corrosion resistance due to their microstructure of short-range order and long-range disordered atomic arrangement [1,2,3,4,5]. As a result, BMGs have tremendous potential as structural materials in aerospace, electronic devices, medical equipment, and other fields [6,7,8]. Among the various types of existing BMGs, Ti-based BMGs have been paid widespread attention owing to their high specific strength, good corrosion resistance, light weight, biocompatibility, and low cost [9,10,11,12]. Ti-based BMGs can be used to manufacture solar wind collectors for absorbing and retaining higher-energy ions [13]. Ti-based BMGs have been applied in preparing Coriolis flow meter sensing tubes, which can be used in chemical industry and semiconductor fields [14]. Additionally, the exceptional mechanical strength of Ti-based BMGs endows them with remarkable load-bearing capacity, an elasticity modulus resembling that of human bone structure, and favorable biocompatibility. These inherent advantages position Ti-based BMG as highly promising materials in the field of biomedical engineering for applications such as medical devices or implants. Liens et al. [15] conducted a comprehensive and exhaustive characterization of Ti_40_Zr_10_Cu_36_Pd_14_ BMG, which was deemed suitable for the fabrication of implants and abutment assemblies or small dimensional pieces for potential dental applications in the future.

However, the critical casting diameter of BMGs used to describe their glass forming abilities (GFAs) seriously limits their practical application [16]. Alloying is an effective method to improve the GFAs of BMGs. Currently, numerous Ti-based BMGs have been developed, including Ti-(Cu, Be) systems [17,18], Ti-Cu-(Ni, Co,) systems [19,20], Ti-Zr-(Cu, Be) systems [17,21], Ti-Zr-Cu-Ni-Be systems [22], and the like. Typical ternary Ti-Zr-Be BMGs with good GFAs can reach the critical diameter of 5 mm [23]. Based on this, Zhao et al. [24,25,26] and Gong et al. [27,28] prepared a series of Ti-Zr-Be-(Ag, Ni, Co, Cu, Fe) quaternary BMGs with good GFAs via an alloying method, of which the maximum critical diameter reached 20 mm. Tang et al. [29] reported a series of (Ti_36.1_Zr_33.2_Ni_5.8_Be_24.9_)_100−*x*_Cu*_x_* BMGs, with the maximum attainable diameter exceeding 50 mm and reaching up to 60 mm. Zhang et al. [30] investigated Ti-Zr-Cu-Fe-Be BMGs, which can achieve a max diameter of up to 50 mm. In addition, in order to avoid toxic metals, biocompatible Ti–Zr–Ta–Si and Ti–Zr–Pd–Si BMGs were manufactured by Oak et al. [31]. These works greatly promoted the application and development of quaternary Ti-based BMGs.

On the other hand, most BMGs have high hardness but lack room temperature plasticity, resulting in catastrophic failure under external loading, making cold work and machining very difficult [32]. The exceptional superplasticity exhibited by BMGs within the supercooled liquid region (SCLR) renders them ideal materials for precise net shaping of various geometries via thermoplastic forming (TPF), overcoming the inherent limitations associated with poor processability of BMGs at ambient temperatures [33]. The TPF of BMGs in the SCLR is controlled by temperature-dependent viscosity and temperature-dependent crystalline [34]. The viscosity of BMGs gradually decreases by several orders of magnitude with increasing temperature in the SCLR, and the processing accuracy can reach the nanometer scale with TPF technology, making BMGs the ideal material for microdevices [32,35]. Gong et al. [36] successfully fabricated Ti_41_Zr_25_Be_30_Fe_5_ nanorods via TPF. Cai et al. [37] successfully developed a 300 μm square-well array pattern and a 5 μm square-hump array on the surface of Ti_40_Zr_10_Cu_34_Pd_14_Sn_2_ using TPF, showcasing the potential for intricate patterning. Additionally, Cai et al. [38] extended this work by fabricating a hierarchical structure on the surface of Ti_40_Zr_10_Cu_34_Pd_14_Sn_2_ BMG through a two-step TPF process, effectively creating 400 nm protrusions atop 2.5 μm square humps. This marked the first instance of a Ti-based BMG achieving a layered structure with both micro-patterns and nano-patterns on the same surface. They further investigated the influence of various TPF conditions on the structural and mechanical properties of Ti_40_Zr_10_Cu_34_Pd_14_Sn_2_ BMG, as well as its compatibility with cellular environments.

However, research on the TPF of Ti-based BMGs is currently limited. To enhance the TPF of BMGs, methods such as incorporating a wetting layer can be employed [39]. Additionally, adjusting the chemical composition of BMGs remains the most effective approach to improve their TPF. Obvious differences of TPF have been observed in different BMGs [34]. At present, there is limited research available on the composition-dependent TPF of Ti-based BMGs. However, TPF requires that the BMGs possess a sufficiently high GFA, as TPF only can be performed after obtaining the BMGs [40]. Therefore, the effect of chemical composition on the TPF of quaternary Ti-based BMGs with high GFA was investigated, which is significant for promoting the application of Ti-based BMGs. In this study, Ti_41_Zr_25_Be_34-*x*_Ni*_x_* (at.%) and Ti_41_Zr_25_Be_34-*x*_Cu*_x_* (at.%) BMG systems were employed to investigate the effect of the change of Ni and Cu content on the thermal stability, TPF, viscosity, and nanoindentation. The correlation between mean atomic distance and hardness is also discussed for Ti_41_Zr_25_Be_34-*x*_Ni*_x_* (at.%) and Ti_41_Zr_25_Be_34-*x*_Cu*_x_* (at.%) BMG systems.

## 2. Experimental Materials and Methods

The master ingots with normal compositions of Ti_41_Zr_25_Be_34*-x*_Ni*_x_* (hereafter, *x* = 4, 6, 8, 10 are denoted as Ni4, Ni6, Ni8, and Ni10, respectively) and Ti_41_Zr_25_Be_34-*x*_Cu*_x_* (hereafter, *x* = 4, 6, and 8 are denoted as Cu4, Cu6, and Cu8, respectively) were fabricated using a KDH-300 induction melting furnace (Henan Kusite Instrument Technology Co., Ltd., Zhengzhou, Henan, China) by arc-melting a blend of high-purity metals (purity ≥ 99.99%) in a Ti-gettered high-purity argon atmosphere. The pure titanium ingot was melted prior to melting the master ingot in order to effectively absorb any residual oxygen. The master ingot, weighing approximately 10 g, was flipped and remelted four times to ensure compositional homogeneity. Cylindrical rods with a 6 mm diameter and a 50 mm length were prepared via a copper mold suction casting method. The amorphous structure of the as-cast samples was checked using a Bruker D8 Advance X-ray diffractometer (Rigaku Industrial Co., Ltd., Tokyo, Japan) with Cu Kα radiation in the 2*θ* range of 10–90° at a 0.02° scanning step and a 3° min^−1^ scanning speed. Differential scanning calorimetry (DSC) tests were performed by a Netzsch STA 449 F3 (NETZSCH-Gerätebau GmbH, Selb, Wunsiedel, Germany) under a flow of purified argon at a 20 K min^−1^ heating rate, using alumina crucibles.

A simple and precise standard, the maximum strain a BMG can undergo in its supercooled liquid state before it eventually crystallizes, was used to characterize the TPF of BMG [34]. The TPF of Ti_41_Zr_25_Be_34*-x*_Ni*_x_* and Ti_41_Zr_25_Be_34-*x*_Cu*_x_* BMG systems characterized by the final diameter *D* was tested according to the standard. Before testing, the top and bottom surfaces of the Ti-based BMG samples were ground using 2000 grit sandpapers. The BMG specimens were positioned between platens and forced against each other with a constant load of 2000 N, using an OTF-1200X-VHP4 hot-pressing machine (Hefei Kejing Material Technology Co., Ltd., Hefei, Anhui, China). The sample volume was about 0.1 cm^3^, the starting processing temperature was glass transition temperature *T*_g_, the constant heating rate was 1 K min^−1^, and the highest processing temperature was chosen as that at which crystallization can still be avoided, in order to maximize the formability during TPF process [41]. The viscosity η of the amorphous alloys in SCLR is a very important parameter, which determines the processing formability [42]. The viscosity of the samples was determined through thermomechanical analyzer (TMA) analysis to further examine the influence of chemical composition on the TPF of Ti-based BMGs. The geometric dimension of viscosity samples was Φ6 mm × 5 mm. The viscosity tests were performed as a function of temperature in the SCLR using TA Q400 TMA (TA Instruments Inc., Wilmington City, DE, USA) with a 10 K min^−1^ heating rate and a 1 N static force. The η of BMGs was determined according to the following equation, as given in [43,44]:

(1) $$\eta =\frac{\sigma_{\mathrm{flow}}}{3\dot{\varepsilon }}$$

where $\dot{\varepsilon }$ denotes the strain rate of the BMGs and *σ*_flow_ is the flow stress of the BMGs. In order to study the relationship between chemical composition and nanoindentation, porosities that remained in the as-cast specimens were excluded to prepare nanoindentation samples via TPF in the SCLR. To control their fictive temperature, all alloys were cooled after TPF from a temperature of ~*T*_g_ + 20 K through the glass transition region at a rate of ~50 K s^−1^.

Prior to conducting the nanoindentation tests, special attention was given to the surface quality of the samples. The samples were ground using 400, 800, 1500, and 2500 grit sequentially, followed by a final polishing step with an alumina paste to achieve a mirror-like finish, and no discernible scratches were observed. Nanoindentation tests were carried out using a U9820A Nano Indenter G200 (Agilent Technologies Inc., Santa Clara City, CA, USA) under the load control mode with a 40 mN peak load for 5 s. Both the loading and unloading rates were set as 1 mN s^−1^. For each sample, the indentation process was repeated five times with the indents spaced 100 μm apart.

## 3. Results

### 3.1. Amorphous Nature

**Figure 1:**
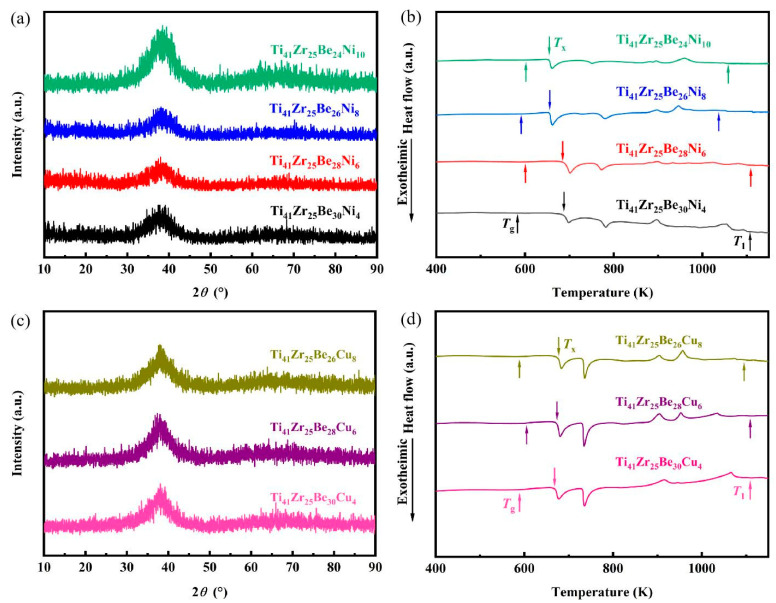
Structure and thermal characterization of Ti_41_Zr_25_Be_30-*x*_Ni*_x_* and Ti_41_Zr_25_Be_30-*x*_Cu*_x_* BMG systems. (**a**) XRD patterns of the Ti_41_Zr_25_Be_34-*x*_Ni*_x_* BMGs with 6 mm rods. *θ:* XRD scattering angle; a.u.: arbitrary unit. (**b**) DSC curves of Ti_41_Zr_25_Be_30-*x*_Ni*_x_* alloys (the heating rate is 20 K min^−1^). (**c**) XRD patterns of the Ti_41_Zr_25_Be_30-*x*_Cu*_x_* BMGs with 6 mm rods. (**d**) DSC curves of Ti_41_Zr_25_Be_34-*x*_Cu*_x_* alloys.

The X-ray diffraction (XRD) patterns of the as-cast materials show that the expected broad amorphous halos with no crystallization were observed (Figure 1a,c). Clear glass transitions and sharp crystallization events were observed in the DSC thermograms (Figure 1b,d), where the *T*_g_ and onset temperature of crystallization *T*_x_ can be identified, confirming the glassy nature of Ti_41_Zr_25_Be_34-*x*_Ni*_x_* and Ti_41_Zr_25_Be_34-*x*_Cu*_x_* BMGs. The width of SCLR, defined as Δ*T* = *T*_x_ − *T*_g_, has often been used to evaluate the thermostability of SCLR [45]. The thermal parameters of Ti_41_Zr_25_Be_34-*x*_Ni*_x_* and Ti_41_Z_r25_Be_34-*x*_Cu*_x_* BMGs are summarized in Table 1. The *T*_g_ decreased from 601 K at Ni4 BMG to 591 K at Ni8 BMG, followed by a slight increase to 595 K at Ni10 BMG. The *T*_g_ of Ti_41_Zr_25_Be_34-*x*_Cu*_x_* BMGs exhibited a decrease followed by a subsequent increase with Cu content. The change trend of *T*_g_ of the Ti_41_Zr_25_Be_34-*x*_Ni*_x_* and Ti_41_Zr_25_Be_34-*x*_Cu*_x_* BMG systems is in good agreement with the data of other researchers [19,22]. The reduced glass transition temperature, *T*_rg_ (=*T*_g_/*T*_l_) [46], has been often quoted to evaluate the GFA of amorphous materials [47]. From Table 1, it can be seen that for the Ti_41_Zr_25_Be_34-*x*_Ni*_x_* alloy series, *T*_rg_ monotonically increased with increasing Ni content from 0.5380 for Ni4 BMG to 0.5716 for Ni10 BMG. For the Ti_41_Zr_25_Be_34-*x*_Cu*_x_* alloy series, *T*_rg_ also monotonically increased with increasing Cu content from 0.5169 for Cu4 BMG to 0.5288 for Cu8 BMG. Δ*H*_m_ represents the crystallization enthalpy and was calculated from the DSC curves (Figure 1b,d). In the Ti-Zr-Be-Ni BMG system, the Δ*H*_m_ decreased from −22.29 J g^−1^ at Ni4 BMG to −34.37 J g^−1^ at Ni6 BMG, followed by increasing to −26.23 J g^−1^ at Ni10 BMG. The Δ*H*_m_ of Cu4, Cu6, and Cu8 BMGs was −25.95 J g^−1^, −35.22 J g^−1^ and −27.00 J g^−1^, respectively.

### 3.2. Thermoplastic Formability

**Figure 2:**
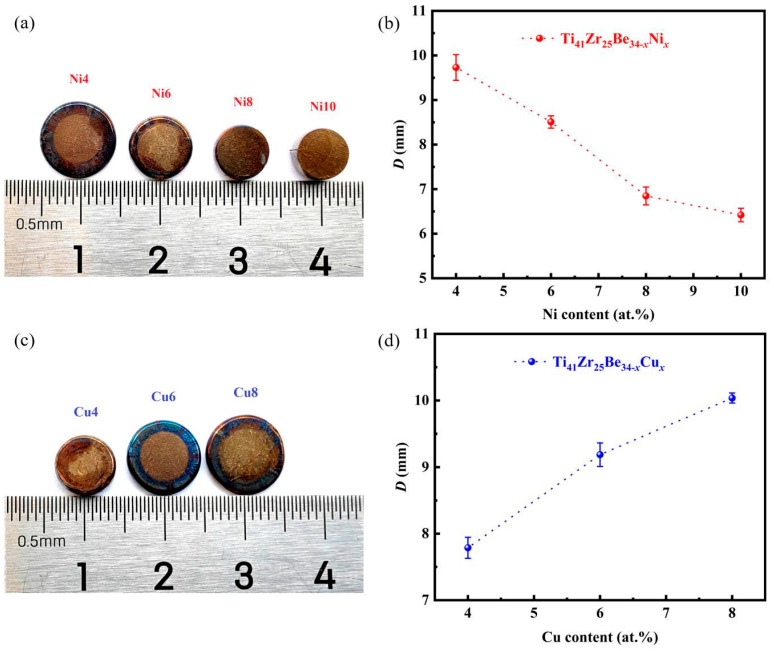
Thermoplastic formability of Ti_41_Zr_25_Be_34*-x*_Ni*_x_* and Ti_41_Zr_25_Be_34-*x*_Cu*_x_* BMG series. (**a**) Top view of the Ti_41_Zr_25_Be_34-*x*_Ni*_x_* samples after the thermoplastic formability characterization evaluation. (**b**) Change of *D* as a function of the Ni content. (**c**) Top view of the Ti_41_Zr_25_Be_34-*x*_Cu*_x_* samples after the thermoplastic formability characterization evaluation. (**d**) Change of *D* as a function of the Cu content.

The TPF of Ti_41_Zr_25_Be_34*-x*_Ni*_x_* and Ti_41_Zr_25_Be_34-*x*_Cu*_x_* BMG series exhibited significant variation depending on the chemical composition (presented in Figure 2). The TPF of Ni4 BMG was the best among Ti_41_Zr_25_Be_34*-x*_Ni*_x_* amorphous alloys and the TPF of Cu8 BMG was the best among Ti_41_Zr_25_Be_34-*x*_Cu*_x_* amorphous alloys. The *D* of the Ti_41_Zr_25_Be_34*-x*_Ni*_x_* alloy system decreased from Ni4 at *D* = 9.73 mm to *D* = 6.42 mm at Ni10; however, the *D* of the Ti_41_Zr_25_Be_34*-x*_Cu*_x_* alloy system increased from Cu4 at *D* = 7.78 mm to *D* = 10.04 mm at Cu8. This indicates that the TPF of Ti_41_Zr_25_Be_34*-x*_Ni*_x_* BMGs decreases with the increase in Ni content; however, the TPF of Ti_41_Zr_25_Be_34-*x*_Cu*_x_* BMGs increases with increasing Cu content. Generally, the width of the SCLR is one of the important indicators of TPF. A wider SCLR not only means better thermal stability of BMGs, but can also better avoid the crystallization of BMGs in the TPF process. Therefore, *S* = Δ*T*_x_/(*T*_l_ − *T*_g_) was presented as a strong indicator of correlation with TPF [27,48,49]. In this sense, a higher value of *S* is indicative of the TPF of the investigated Ti-based BMGs to a certain extent. As illustrated in Table 1, the Cu8 alloy possessed a larger *S* value of 0.1844 compared with other studied Ti-Zr-Be-(Ni, Cu) BMGs, and its *D* value (10.04 mm) also ranked highest among the Ti-Zr-Be-(Ni, Cu) BMGs.

### 3.3. Viscosity

**Figure 3:**
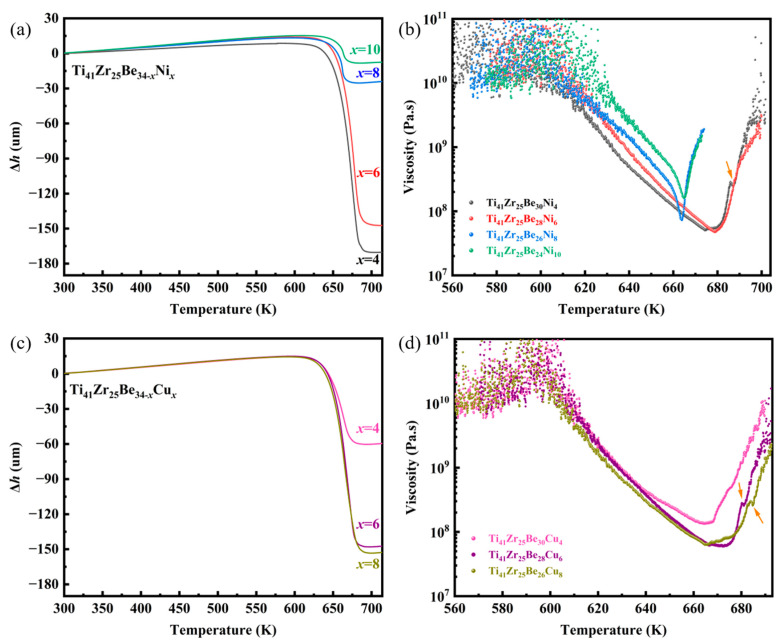
Viscosity as a function of temperature of the Ti-based BMGs. (**a**) TMA curves of Ti_41_Zr_25_Be_34-*x*_Ni*_x_*. (**b**) Measured viscosity of the Ti_41_Zr_25_Be_34-*x*_Ni*_x_* BMGs in the SCLR. (**c**) TMA curves of Ti_41_Zr_25_Be_34-*x*_Cu*_x_*. (**d**) Measured viscosity of the Ti_41_Zr_25_Be_34-*x*_Cu*_x_* BMGs in the SCLR.

The TMA results of the investigated Ti_41_Zr_25_Be_34-*x*_Ni*_x_* and Ti_41_Zr_25_Be_34-*x*_Cu*_x_* BMG series are presented in Figure 3. The TMA curves exhibit a decrease in compressive deformation (Δ*h*) with increasing Ni content in the Ti_41_Zr_25_Be_34-*x*_Ni*_x_* BMG system (Figure 3a), while an increase in Δ*h* can be observed with the increase in Cu content in the Ti_41_Zr_25_Be_34-*x*_Cu*_x_* BMG system (Figure 3c). Ti_41_Zr_25_Be_30_Ni_4_ BMG presents the largest Δ*h* with 174.0 um and Ti_41_Zr_25_Be_26_Cu_8_ BMG exhibits the largest Δ*h* with 153.3 um. The value of Δ*h* also indicates the TPF of the BMG samples in the SCLR. For example, Pan Gong et al. [27] used Δ*h* to characterize the TPF of Ti-Zr-Be-Co BMGs. In order to investigate the influence of key parameters on the TPF, the TMA results were converted into a plot of viscosity versus temperature via Equation (1). The variation of viscosity with temperature in the SCLR for Ti_41_Zr_25_Be_34-*x*_Ni*_x_* and Ti_41_Zr_25_Be_34-*x*_Cu*_x_* BMGs is depicted in Figure 3b and 3d, respectively. The viscosity of Ti_41_Zr_25_Be_34-*x*_Ni*_x_* and Ti_41_Zr_25_Be_34-*x*_Cu*_x_* BMGs exhibits a significant variation with a pronounced decrease by several orders of magnitude at the *T*_g_ temperature and an increase as it approaches the *T*_x_ temperature.

The viscosity includes secondary drops (indicated by the arrow), which can be observed in the Ni4, Cu6, and Cu8 alloys. This phenomenon was also observed by Parthiban et al. [50] and Stoica et al. [51], who reported that the investigated Fe-Co-B-Si-Nb-Cu BMGs presented two glass-transition-like events. The above case may indicate that after the initial crystallization, the samples exhibit a structure comprising a crystalline phase along with the remaining amorphous matrix.

### 3.4. Nanoindentations

**Figure 4:**
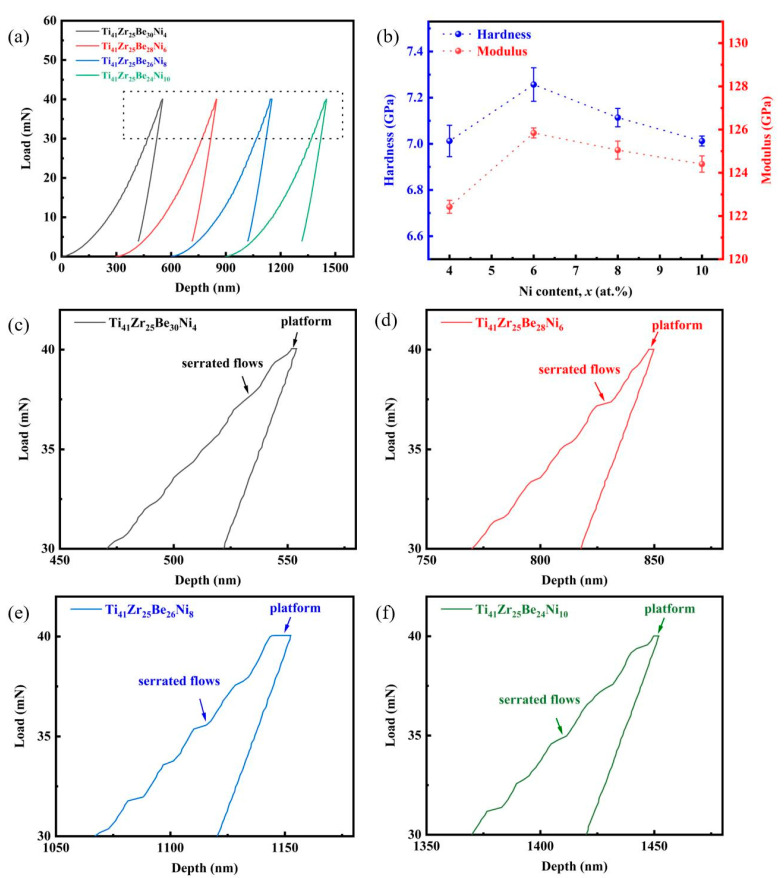
Nanoindentation results of Ti_41_Zr_25_Be_34-*x*_Ni*_x_* BMG system. (**a**) Representative *P*-*h* curves of Ti_41_Zr_25_Be_34-*x*_Ni*_x_* BMGs at a constant loading rate of 1 mN s^−1^; (**b**) relationship between hardness, modulus, and Ni content; (**c**–**f**) a close view of the rectangular dotted zone in Figure 4a.

**Figure 5:**
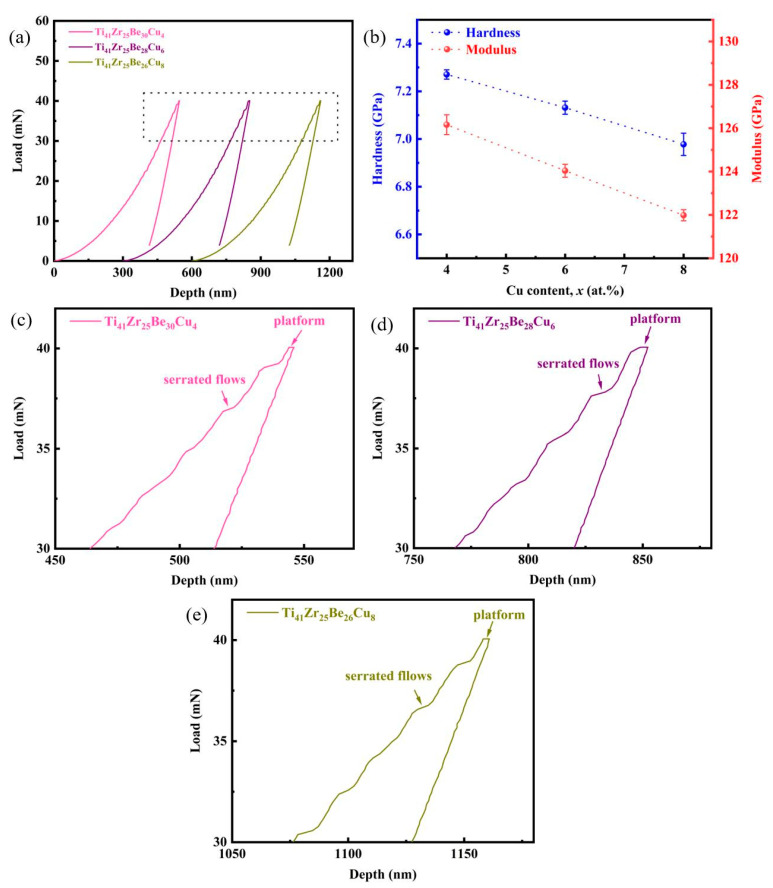
Nanoindentation results of Ti_41_Zr_25_Be_34-*x*_Cu*_x_* BMG system. (**a**) Representative *P*-*h* curves of Ti_41_Zr_25_Be_34-*x*_Cu*_x_* BMGs at a constant loading rate of 1 mN s^−1^; (**b**) relationship between hardness, modulus, and Cu content; (**c**–**e**) a close view of the rectangular dotted zone in Figure 5a.

The typical load–displacement (*P*-*h*) curves of Ti_41_Zr_25_Be_34*-x*_Ni*_x_* and Ti_41_Zr_25_Be_34-*x*_Cu*_x_* BMG systems are given in Figure 4a and Figure 5a, respectively. To obtain a comprehensive analysis of the loading part, each *P*-*h* curve has been horizontally shifted by some distance to the right, and the *P*-*h* curves present the same change trend. The rectangular dotted zone in Figure 4a is magnified to provide a close-up view of the *P*-*h* curves of Ti_41_Zr_25_Be_34*-x*_Ni*_x_* BMGs, as shown in Figure 4c–f, and the rectangular dotted zone in Figure 5a is also magnified to provide a close-up view of the *P*-*h* curves of Ti_41_Zr_25_Be_34*-x*_Cu*_x_* BMGs, as shown in Figure 5c–e. For Ti_41_Zr_25_Be_34*-x*_Ni*_x_* and Ti_41_Zr_25_Be_34-*x*_Cu*_x_* BMG systems, the serrated flows and platforms in the *P*-*h* curves are observed in the magnified zones of the *P*-*h* curves. The platform continues to deform even after reaching maximum load, indicating the occurrence of creep as the indentation depth further increases under maximum load [52]. Subsequently, during unloading, elastic recovery is observed with a decrease in indentation depth. The occurrence of serrated flows in the loading curves is attributed to the propagation of shear bands [53]. The nanoindentation tests induced both plastic and elastic deformation in the samples. The occurrence of serrated flows is associated with localized plastic flow of BMGs, indicating the activation of shear bands [54,55]. 

The results for the hardness (*H*) and elastic modulus (*E*) of Ti_41_Zr_25_Be_34*-x*_Ni*_x_* and Ti_41_Zr_25_Be_34-*x*_Cu*_x_* BMG series calculated using the Oliver–Pharr method [56] are shown in Figure 4b and Figure 5b, respectively. The *H* of Ti_41_Zr_25_Be_34*-x*_Ni*_x_* BMGs first increased from Ni4 BMG with *H* = 7.01 GPa to *H* = 7.26 GPa at Ni6 BMG, and then decreased with further increasing Ni content to *H* = 7.03 GPa for Ni10 BMG. The *E* of Ti_41_Zr_25_Be_34*-x*_Ni*_x_* BMGs initially increased with Ni content and reached a maximum of *H* = 125.84 GPa at Ni6 BMG, and then decreased with increasing Ni content. The change trend of *E* for the Ti_41_Zr_25_Be_34*-x*_Ni*_x_* BMGs is the same as that of *H* for Ti_41_Zr_25_Be_34*-x*_Ni*_x_* BMGs, while both the *H* and *E* of Ti_41_Zr_25_Be_34-*x*_Cu*_x_* BMGs decreased with an increase in Cu content (Figure 5b). 

## 4. Discussion

**Figure 6:**
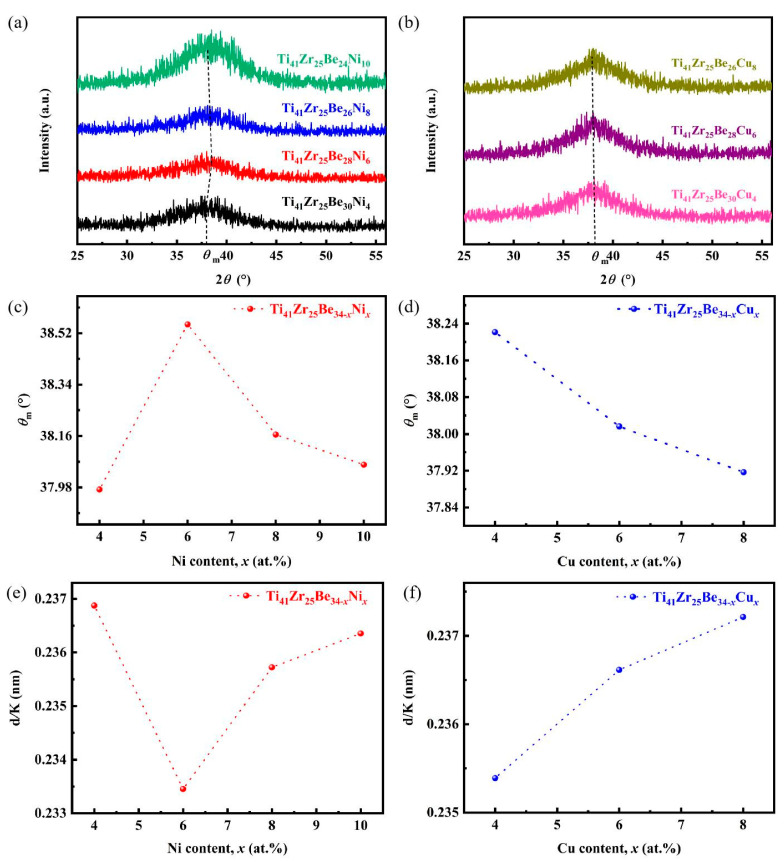
Relationship of *θ*_m_ and d/K with the change of chemical composition in Ti_41_Zr_25_Be_34*-x*_Ni*_x_* BMGs and Cu content of Ti_41_Zr_25_Be_34-*x*_Cu*_x_* BMG systems. (**a**) Magnified maximal diffracted intensity in XRD pattern of Ti_41_Zr_25_Be_34*-x*_Ni*_x_* BMGs. (**b**) Magnified maximal diffracted intensity in XRD pattern of Ti_41_Zr_25_Be_34-*x*_Cu*_x_* BMGs. (**c**) Change of *θ*_m_ as a function of Ni content for Ti_41_Zr_25_Be_34*-x*_Ni*_x_* BMGs. (**d**). Change of *θ*_m_ as a function of Cu content for Ti_41_Zr_25_Be_34-*x*_Cu*_x_* BMGs. (**e**) The correlation between the d/K and Ni content for Ti_41_Zr_25_Be_34*-x*_Ni*_x_* BMGs. (**f**) The correlation between the d/K and Cu content for Ti_41_Zr_25_Be_34-*x*_Cu*_x_* BMGs.

Enlarging the maximal diffracted intensity in Figure 1a,c, it can be observed that the angle at maximal diffracted intensity *θ*_m_ of Ti_41_Zr_25_Be_34*-x*_Ni*_x_* BMGs first increased with the increase in Ni content, reached a peak at Ni6 BMG, and then decreased with an increase in Ni content (Figure 6a). The *θ*_m_ of Ti_41_Zr_25_Be_34-*x*_Cu*_x_* BMGs decreased with increasing Cu content (Figure 6b). The position of *θ*_m_ was determined through simulation using the Gaussian model. Even though no quantitative analysis of the average atomic distance was tried, the model for diatomic gas introduced by Guinier [57] was utilized to monitor the alterations in the relative atomic structure of the studied alloys as their chemical composition changed. Hufnagel et al. [58], Caron et al. [59], Supriya et al. [60], Yang et al. [61], Sharma et al. [62], and Lv et al. [63] also applied this model to study the atomic structural changes in BMGs subjected to elastic stress and established a connection between the atomic distance of a diatomic gas molecule and the Bragg diffraction angle, via the following equation:

1.23*λ* = 2*d*sin*θ* (2)

where *λ* is the wavelength of the radiation and *d* denotes the atomic distance of a diatomic gas. In Equation (2), the factor 1.23 is a result of the random orientation of molecules within the gas [57,62]. Initially, as a simplification, we can assume a similar relationship for the average atomic distance within an amorphous solid, replacing 1.23 with a factor *K*. Then, we can make the preliminary assumption that *K* remains constant over the entire concentration range under investigation and thus assess the relative alteration of the average atomic distance with a change in chemical composition.

The *θ*_m_ of Ti_41_Zr_25_Be_34*-x*_Ni*_x_* alloys initially exhibited a slight increase and subsequently decreased with increasing Ni content (Figure 6c), resulting in a decrease followed by an increase in the value of d/K with increasing Ni content (Figure 6e). This implies that the mean atomic distance first decreased and then increased with increasing Ni content, and also indicates that then atomic density underwent an initial rise followed by a subsequent decline. The mass density increased from 5.07 g/cm^3^ at Ni4 BMG to 5.138 g/cm^3^ at Ni6 BMG, followed by a slight decrease to 5.072 g/cm^3^ at Ni10 BMG. The mass density of the samples prepared for nanoindentation tests was determined using Archimedes’ principle. In the Ti_41_Zr_25_Be_34*-x*_Ni*_x_* BMG system, the mean atomic distance exhibited an inverse relationship with mass density. As a result, hardness initially increased and then further decreased with increasing Ni content (Figure 4b). When the Ni content was at 6 at.% in the Ti_41_Zr_25_Be_34*-x*_Ni*_x_* BMG system, the d/K value reached its minimum, indicating that Ni6 possessed a more densely packed atomic structure and consequently exhibited the highest hardness (7.26 GPa). Following the same underlying principle, the *θ*_m_ decreased with an increase in Cu content in the Ti_41_Zr_25_Be_34*-x*_Cu*_x_* alloys (Figure 6d), leading to an increase in d/K with increasing Cu content (Figure 6f). Figure 6f also illustrates that, in the as-cast state, the mean atomic distance of Ti_41_Zr_25_Be_34-*x*_Cu*_x_* BMGs tended to increase as the Cu content increased, accordingly resulting in a decrease in atomic density. The mass density of Ti_41_Zr_25_Be_34-*x*_Cu*_x_* BMGs exhibited a gradual decrease from 5.04 g/cm^3^ at Cu4 BMG to 5.02 g/cm^3^ at Cu8 BMG. In the Ti_41_Zr_25_Be_34*-x*_Cu*_x_* BMG system, the mean atomic distance also exhibited an inverse relationship with mass density. Thus, the hardness decreased with an increase in Cu content. At 4 at.% Cu in the Ti_41_Zr_25_Be_34*-x*_Cu*_x_* BMG system, Cu4 had the maximum value of hardness (7.27 GPa).

## 5. Conclusions

Two BMG systems of Ti-based Ti_41_Zr_25_Be_34-*x*_Ni*_x_* and Ti_41_Zr_25_Be_34-*x*_Cu*_x_* BMGs were prepared using a copper mold suction casting method and the effect of Ni and Cu content on the thermoplastic formability, viscosity, and nanoindentation was studied. The current work is summarized as follows:

1. The viscosity of Ti-based BMGs exhibited a gradual decrease of several orders of magnitude with increasing temperature in the SCLR, and the TPF is influenced by both the viscosity and the width of the SCLR. In Ti_41_Zr_25_Be_34-*x*_Ni*_x_* BMGs, the TPF decreased with increasing Ni content, and Ti_41_Zr_25_Be_30_Ni_4_ exhibited the highest TPF compared with the other alloys due to its wider SCLR and lower values of viscosity in the SCLR. Conversely, for Ti_41_Zr_25_Be_34-*x*_Cu*_x_* BMGs, the TPF was enhanced with increasing Cu content, reaching its peak at 8 at.% Cu (Ti_41_Zr_25_Be_26_Cu_8_). This result is similarly ascribed to its wider SCLR and lower viscosity in the SCLR.
2. The nanoindentation tests reveal that the hardness and modulus of Ti_41_Zr_25_Be_34-*x*_Ni*_x_* BMGs exhibited an initial increase followed by a decrease with increasing Ni content. Specifically, at 6 at.% Ni, the highest values of hardness (7.26 GPa) and modulus (125.8 GPa) were observed. Furthermore, the hardness and modulus of Ti_41_Zr_25_Be_34-*x*_Cu*_x_* decreased with increasing Cu content. At 4 at.% Cu, the alloy demonstrated the maximum values of hardness (7.27 GPa) and modulus (126.2 Gpa) among the Ti_41_Zr_25_Be_34-*x*_Cu*_x_* BMGs. In this study, based on Bragg’s law, the position of the maximal diffracted intensity *θ*_m_ was utilized to reflect the mean atomic distance, suggesting the variation in atomic density. This indicates the varying tendency of the hardness of Ti-based BMGs.

## Figures and Tables

**Table 1:** Glass transition temperature (*T*_g_), onset temperature of crystallization (*T*_x_), crystallization exothermic heat (Δ*H*), melting temperature (*T*_m_), and liquid temperature (*T*_l_) of Ti_41_Zr_25_Be_30-*x*_Ni*_x_* and Ti_41_Zr_25_Be_34-*x*_Cu*_x_* BMGs obtained via DSC at a heating rate of 20 K min^−1^, Δ*T*_x_ = *T*_x_ − *T*_g_, *T*_rg_ = *T*_g_/*T*_l_, *S* = Δ*T*_x_/(*T*_l_ − *T*_g_).

Composition	*T*_g_ (K)	*T*_x_ (K)	Δ*T*_x_ (K)	*T*_m_ (K)	*T*_l_ (K)	*T*_rg_	Δ*H*_m_ (J g^−1^)	*S*
Ti_41_Zr_25_Be_30_Ni_4_	601	684	83	876	1118	0.5380	−22.29	0.1605
Ti_41_Zr_25_Be_28_Ni_6_	597	680	83	868	1108	0.5388	−34.37	0.1624
Ti_41_Zr_25_Be_26_Ni_8_	591	655	64	874	1050	0.5629	−33.23	0.1394
Ti_41_Zr_25_Be_24_Ni_10_	595	654	59	864	1041	0.5716	−26.23	0.1323
Ti_41_Zr_25_Be_30_Cu_4_	579	669	90	870	1120	0.5169	−25.95	0.1664
Ti_41_Zr_25_Be_28_Cu_6_	590	674	84	881	1119	0.5273	−35.22	0.1588
Ti_41_Zr_25_Be_26_Cu_8_	578	673	95	885	1093	0.5288	−27.00	0.1845

## Data Availability

Data are contained within the article.
